# Novel Cul3 binding proteins function to remodel E3 ligase complexes

**DOI:** 10.1186/1471-2121-15-28

**Published:** 2014-07-10

**Authors:** Wananit Wimuttisuk, Mark West, Brittney Davidge, Kebing Yu, Arthur Salomon, Jeffrey D Singer

**Affiliations:** 1 Department of Biology, Portland State University, Portland, Oregon, USA; 2 Department of Molecular Biology, Cell Biology and Biochemistry, the Center for Genomics and Proteomics Brown University, Providence, RI, USA; 3 Department of Chemistry, Brown University, Providence, RI, USA; 4 Present address: National Center for Genetic Engineering and Biotechnology (BIOTEC), Bangkok, Thailand

**Keywords:** Cullin3, Tandem-affinity purification, BTB domain-containing protein, BCR ubiquitin ligase complex, Mass spectrometry, E3 ubiquitin ligase, Protein purification, Ubiquitin, Ubiquitin ligase

## Abstract

**Background:**

Cullins belong to a family of scaffold proteins that assemble multi-subunit ubiquitin ligase complexes to recruit protein substrates for ubiquitination via unique sets of substrate adaptor, such as Skp1 or Elongin B, and a substrate-binding protein with a conserved protein-protein interacting domain, such as *l*eucine-*r*ich *r*epeats (LRR), a WD40 domain, or a zinc-finger domain. In the case of the Cullin3 (Cul3), it forms a *B*TB-*C*ul3-*R*bx1 (BCR) ubiquitin ligase complex where it is believed that a BTB domain-containing protein performs dual functions where it serves as both the substrate adaptor and the substrate recognition protein.

**Results:**

Tandem affinity purification and LC/MS-MS analysis of the BCR complex led to the identification of 10,225 peptides. After the SEQUEST algorithm and CDART program were used for protein identification and domain prediction, we discovered a group of *C*ul3-bound proteins that contain either the *L*RR or *W*D40 domain (CLWs). Further biochemical analysis revealed that the LRR domain-containing CLWs could bind both Cul3 and BTB domain-containing proteins. The dual binding role for the LRR domain-containing CLWs results in causing the BTB-domain protein to become a substrate instead of an adaptor.

To further distinguish potential substrates from other components that are part of the BCR ubiquitin ligase complex, we altered the parameters in the SEQUEST algorithm to select for peptide fragments with a modified lysine residue. This method not only identifies the potential substrates of the BCR ubiquitin ligase complex, but it also pinpoints the lysine residue in which the post-translational modification occurs. Interestingly, none of the CLWs were identified by this method, supporting our hypothesis that CLWs were not potential substrates but rather additional components of the BCR ubiquitin ligase complex.

**Conclusion:**

Our study identified a new set of Cul3-binding proteins known as CLWs via tandem affinity purification and LC/MS-MS analysis. Subsequently, our biochemical analysis revealed that some CLWs modify binding of BTB domain-containing proteins to the complex, causing degradation of the BTB domain-containing protein. As these CLWs were excluded from our list of substrates, we propose that CLWs serve as unique Cul3 binding proteins that provide an alternative regulatory mechanism for the complex.

## Background

Seven cullins have been identified in mammalian cells (Cul1, 2, 3, 4A, 4B, 5, and 7); each cullin assembles a unique set of ubiquitin ligase complexes that catalyze the formation of polyubiquitin chains to signal the degradation of target proteins by the ubiquitin-dependent proteolytic pathway [1,2]. The three best-characterized cullins, Cul1, Cul2, and Cul3, form diverse groups of ubiquitin ligase complexes with different sets of substrate recognition modules. For instance, Cul1 forms an *S*kp1-*C*ullin-*F*-box (SCF) complex that acquires substrate specificity via two proteins: a Skp1 linker protein and a substrate adaptor protein containing an F-box domain [3,4]. Likewise, Cul2 forms an *E*longin-*C*ul2-*S*OCS-box (ECS) ubiquitin ligase complex with a ubiquitin-like protein Elongin B, a Skp1-like linker protein Elongin C and a substrate adaptor SOCS-box containing protein [5,6]. Conversely, Cul3 forms a *B*TB-*C*ul3-*R*bx1 (BCR) ubiquitin ligase complex, in which a BTB domain-containing protein serves as both a linker and a substrate adaptor module [7-9]. It is estimated that more than 200 BTB domain-containing proteins are expressed in human cells, which could accommodate a very large number of substrates for the BCR ubiquitin ligase complex [9].

Previous studies have uncovered new substrates and unique components of cullin ubiquitin ligase complexes that suggest new mechanisms for substrate recruitment [8,10-13]. For example, DDB1 was originally identified as a substrate adaptor that bound to the N-terminus of the Cul4A complex using its one β-propeller domain (BPB), while utilizing the double-β-propeller domain (BPA-BPC) to provide substrate specificity for the Cul4A ubiquitin ligase complex [14]. However, a study using mass spectrometry analysis of the Cul4A complex has identified a group of WD40 domain-containing proteins called DWD that can bind to both Cul4A and DDB1. These DWD proteins were later shown to serve as additional substrate adaptors for the Cul4A complex [12,13]. Because of the functional similarity between DDB1 and BTB domain-containing proteins, this finding suggests a possibility that the substrate adaptor component of the BCR complex may not be limited to BTB domain-containing proteins.

Here we performed tandem affinity purification (TAP) followed by *Mu*lti*d*imensional *P*rotein *I*dentification *T*echnology (MudPIT) mass spectrometry to identify new components of the BCR complex. Several known Cul3-binding proteins and substrates of the BCR ubiquitin ligase complex were identified by this method. In an attempt to rapidly analyze the potential of the BCR ubiquitin ligase complex from this list of proteins, we modified the parameters in the SEQUEST algorithm so that it would recognize the peptide sequence with a lysine residue that is attached to two glycine residues on its side chain, which is indicative of a protein that has been conjugated to ubiquitin or a ubiquitin-like molecule prior to the tryptic digest.

In addition to many known Cul3 binding proteins and new substrates, identified by remnants of conjugated ubiquitin, we have identified two classes of *C*ul3-bound proteins with either *L*eucine-rich repeats (LRR) or a *W*D40 domain, which will be referred to as CLWs in this study.

## Results

### Tandem affinity purification of *in vivo* Cul3 complexes

**Figure 1:**
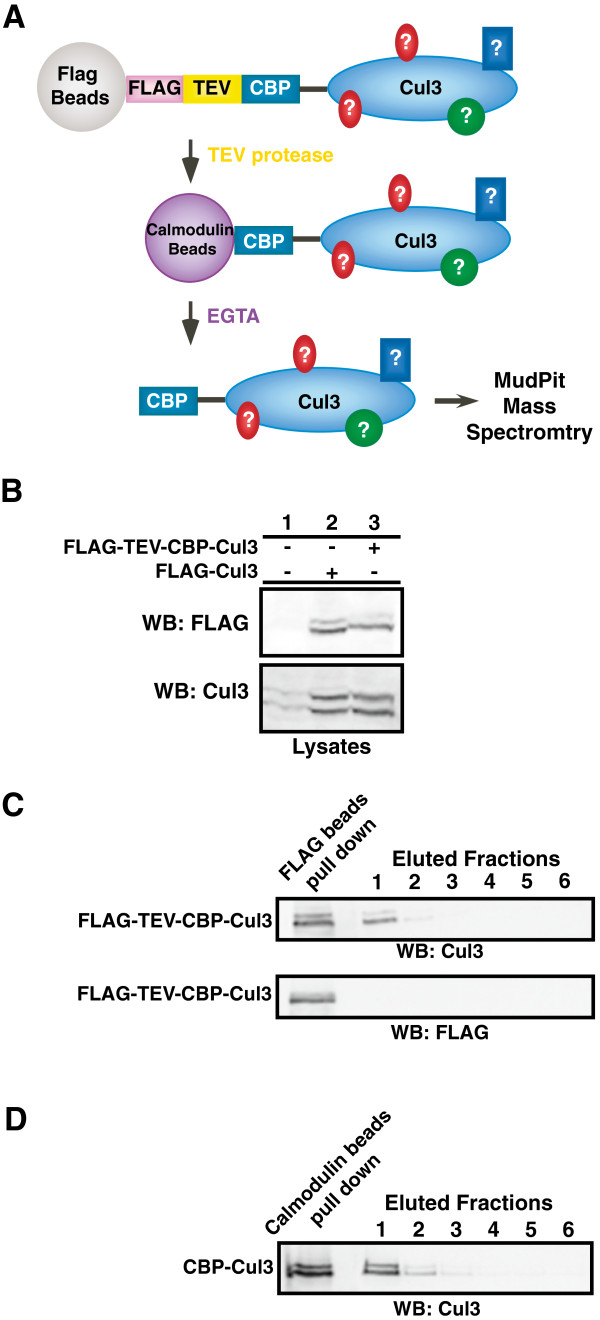
**Tandem affinity purification of 3XFLAG-TEV-CBP-Cul3 complexes. A**. Diagram for the tandem affinity purification of the Cul3 binding proteins. 3XFLAG-TEV-CBP-Cul3 was first affinity purified using anti-FLAG M2 affinity gel resin. Subsequently, the Cul3 complexes were cleaved off the gel resin by the TEV protease enzyme. The eluted CBP-Cul3 complexes were affinity purified using calmodulin-conjugated beads and the Cul3 complexes were subsequently subjected to analysis by MudPIT mass spectrometry. **B**. Untransfected HEK293 cells (lane 1) and HEK293 cells transfected with either FLAG-tagged Cul3 (lane 2) or FLAG-TEV-CBP-Cul3 (lane 3) were harvested and sonicated in RIPA buffer. Cell lysates were immunoblotted and probed with anti-FLAG (top panel) and anti-Cul3 antibodies (bottom panel). **C**. Complexes containing Cul3 were isolated in the first step of the tandem affinity purification. Cell lysates containing FLAG-TEV-CBP-Cul3 protein were subjected to immunoprecipitation using anti-FLAG beads (left lane). CBP-Cul3 was released from the beads using the TEV protease (eluted fractions, lane 1). The FLAG beads were subsequently washed with TEV protease buffer (eluted fractions, lanes 2–4) and calmodulin-binding buffer (eluted fractions, lanes 5–6). The collected supernatants were immunoblotted and probed with anti-Cul3 antibody (top panel) and anti-FLAG antibody (bottom panel). **D**. The CBP-Cul3 complex was isolated in the second step of the tandem affinity purification. Ten milliliters of combined CBP-Cul3 fractions from the first step of the tandem affinity purification were incubated with calmodulin-conjugated beads, followed by several washes with the calmodulin-binding buffer and the calmodulin-rinsing buffer. A sample of protein-bound calmodulin beads was collected (left lane). Cul3 complexes were eluted from the calmodulin beads using the calmodulin-eluting buffer containing EGTA (lane 1–6). The eluted fractions were immunoblotted and probed with anti-Cul3 antibody. The fractions that contained the most Cul3 were selected for MudPIT analysis. See Additional file 3: Table S3.

In this study, a combination of tandem affinity purification (TAP) and MudPIT mass spectrometry were used to identify new members of the BCR ubiquitin ligase complex. To obtain highly purified Cul3 complexes, an expression cassette encoding a FLAG-TEV-CBP-Cul3 fusion protein was constructed so that the Cul3 complex could be purified using anti-FLAG Sepharose beads and calmodulin-conjugated beads (Figure 1A). The size and expression levels of the FLAG-TEV-CBP-Cul3 fusion protein were verified by immunoblotting and probing against either anti-Cul3 antibody or anti-FLAG antibody (Figure 1B). As the proportion of neddylated to unneddylated Cul3 was roughly the same in endogenous Cul3 and overexpressed Cul3 (Figure 1B, bottom panel, lane 1 and 3), we reasoned that these purified Cul3 complexes would likely to be part of a functional BCR ubiquitin ligase complex rather than a free Cul3 monomer, and thus we proceeded with our experiment. The Cul3 complexes were purified with anti-FLAG Sepharose beads and TEV protease cleavage, where the lack of protein bands in the FLAG immunoblot confirmed that the FLAG-tag had been cleaved off the eluted Cul3 complexes (Figure 1C). Subsequently, the CBP-Cul3 complexes were purified with calmodulin-conjugated beads (Figure 1D).

### Data analysis on the MS/MS spectra and potential binding proteins of the BCR ubiquitin ligase complex

Five independent MudPIT experiments were performed for the Cul3 fusion protein as well as one actin control. The SEQUEST algorithm was set to match the experimentally obtained mass spectra with theoretical peptides from the human NCBI nr database, revealing a total of 5,964 peptides from the purified actin control (Table 1, top) and 10,225 peptides from the purified Cul3 complexes (Table 1, bottom) [15,16]. Low quality spectra with a mass error greater than 20 ppm, low Xcorr values (charge 1 < 2, charge 2 < 2.5, and charge 3 < 3), and other known contaminants were discarded. The resulting peptides were categorized into two groups based on the number of peptide sequences that match the predicted protein. The first group of Cul3-binding protein includes proteins that were either identified by at least two different peptide sequences or known Cul3-binding proteins that were identified with at least one peptide sequence in this study, such as CAND1, Nedd8, KLHL5, Rpn1, and Rpn5 (Additional file 1: Table S1). The second group of potential Cul3-binding proteins was identified via mass spectra of one peptide sequence, which required additional manual validation of the MS/MS spectra as well as further biochemical analysis to confirm their interactions with Cul3 complexes (Additional file 2: Table S2). This list includes proteins that may be involved in the ubiquitination pathway, such as pVHL-interacting deubiquitinating enzyme 1, STIP1 homology and U-box containing protein 1, and ubiquitin specific proteases.

**Table 1:** Summary of TAP tag experiments to find Cul3 and actin-binding proteins

**Exp. #**	**# of cells**	**Total protein (mg)**	**TAP yield (mg)**	**Separation**	**MS input (mg)**	**Peptides**	**Peptides </=20 ppm error**
**Actin-binding proteins bound to 3XFLAG-TEV-CBP-Actin:**							
1	9.5 × 10^7^	264	64	MudPIT	15	5964	883
**Cul3-binding proteins bound to 3XFLAG-TEV-CBP-Cul3:**							
1	6.9 × 10^7^	192	280	MudPIT	15	4158	562
2	6.9 × 10^7^	192	52	MudPIT	15	1395	234
3	7.5 × 10^7^	208	36	MudPIT	15	1862	393
4	6.5 × 10^7^	180	39	MudPIT	15	1405	276
5	6.5 × 10^7^	180	39	MudPIT	15	1405	117
			**Total Cul3 bound peptides:**			**10225**	**1582**

See also Additional file 1: Table S1.

### Identification of leucine-rich repeats and WD40 domain-containing proteins as Cul3 binding proteins

Proteins that corresponded to the identified peptide spectra were subjected to the domain prediction program CDART and categorized based on conserved domains [17]. When compared with the list of proteins identified from purified actin complexes, we observed enrichment for proteins that contain WD40 domains and leucine-rich repeats (LRR) in the purified Cul3 data set with the percent enrichment scores of 73.3% of WD40 domain and 90.9% for LRR domain-containing proteins in the Cul3-bound complex in contrast to 26.7% of WD40 domain and 9.1% of the LRR domain-containing protein in the actin-bound complex (Additional file 3: Tables S3 and Additional file 4: Table S4). Because domain enrichment maybe indicative of preferential binding between the BCR complex and proteins with LRR/WD40 domains, we chose to further explore the role of these LRR/WD40 domains in the mammalian BCR complex.

### The leucine-rich repeats (LRR) and WD40 domain-containing proteins (CLWs) bind Cul3

**Figure 2:**
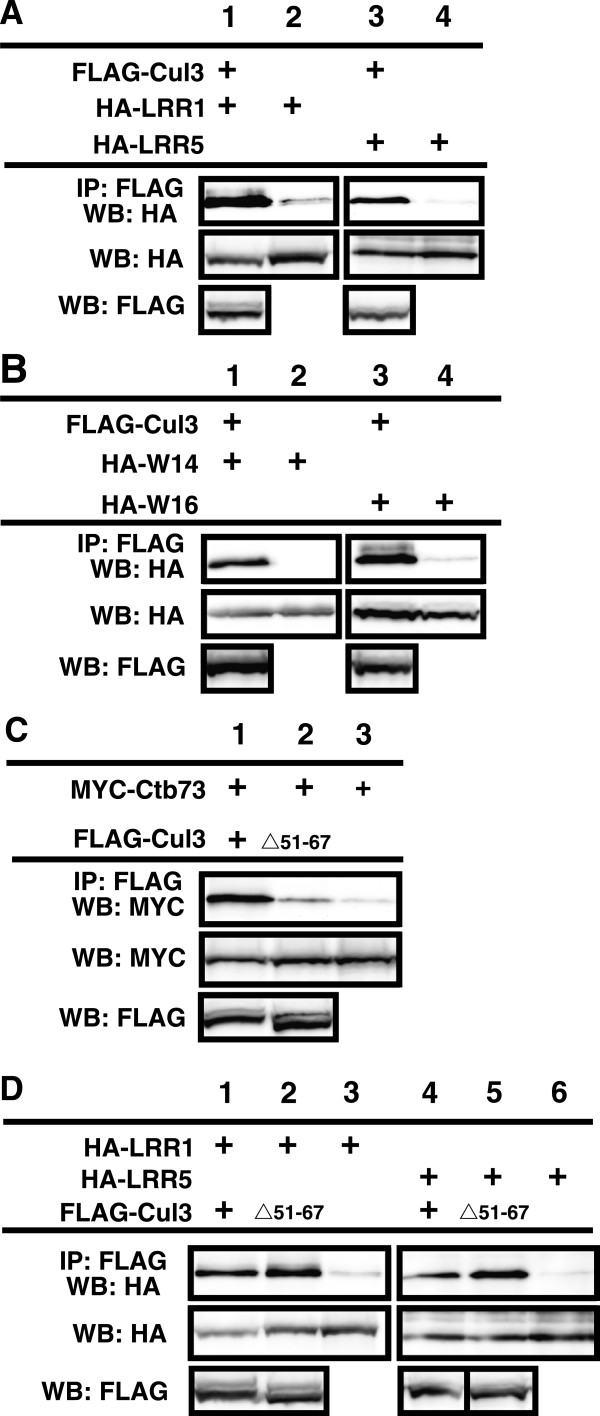
**The newly identified Cul3 binding proteins (CLWs) bind to Cul3 in cells.** HEK293 cells were transfected with vectors expressing FLAG-tagged Cul3 and HA-tagged CLWs. **A**. Western blot analysis shows expression and binding of Cul3 and two LRR domain-containing proteins, LRR1 (left two lanes) and LRR5 (right two lanes). **B**. Western blot analysis shows expression and binding of Cul3 and two WD40 domain-containing proteins, W14 (left two lanes) and W16 (right two lanes). **C**. Wild type Ctb73 was cotransfected with wild type Cul3 (first lane), or a Cul3Δ51-67 mutant (second lane) or expressed alone (third lane). Upper panel shows relative binding between Cul3 and Ctb73, while lower panel shows the expression levels of Ctb73. **D**. Immunoprecipitation assays to detect binding between two different LRR proteins: LRR1 (lane 1–3) and LRR5 (lane 4–6). Wild type Cul3 was expressed in lanes 1 and 4, while the Cul3Δ51-67 mutant was expressed in lane 2 and 5. Lastly, Cul3 was not transfected in lane 3 and 6, which serves as negative controls for the immunoprecipitation assays. See also, Additional file 4: Table S4.

Three leucine-rich repeat proteins (LRRs, LRR1, LRR3 and LRR5 (fibromodulin)) and two WD40 domain-containing proteins (W14 and W16) were cloned from the list of 32 MudPIT-identified *C*ul3-bound proteins with *L*RR or *W*D40 domains (CLWs) to verify their binding to Cul3. The cDNAs were fully sequenced and cloned into mammalian expression vectors in frame with the HA-tag. The proteins were co-expressed with FLAG-tagged Cul3 (FLAG-Cul3). Immunoprecipitation assays revealed that FLAG-Cul3 was able to specifically bind the HA-tagged CLWs with either the LRR (Figure 2A) or the WD40 domains (Figure 2B), which further confirms the results from the proteomic analysis.

### Cul3 mutants that cannot bind BTB domain-containing proteins have enhanced binding to LRRs

To clarify how other Cul3 binding proteins affect or regulate the binding of LRRs to Cul3, we tested a variety of Cul3 mutants that were unable to bind various Cul3 binding partners. Mutants that could not be neddylated (K712R) or could not bind RBX1 (F665D) were able to bind the LRR proteins in an identical fashion to wild type Cul3 (not shown). Surprisingly, mutants that could not bind BTB domain-containing proteins (Δ51-67, Figure 2C) showed enhanced binding to both LRR1 and LRR5 (Figure 2D). This observation led us to investigate a possible interaction between LRRs and BTB domain-containing proteins.

### LRRs bind to BTB domain-containing proteins

**Figure 3:**
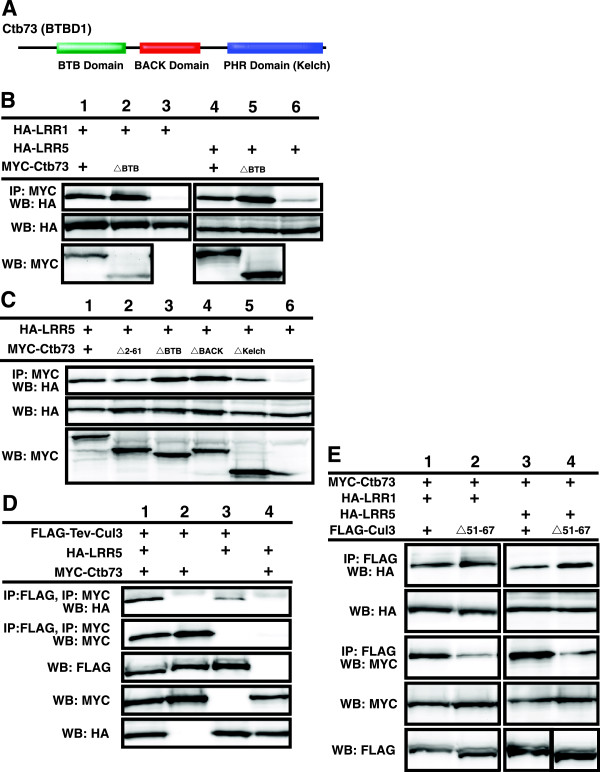
**The BTB domain-containing proteins bind LRRs, while the ΔBTB mutant enhances the binding interaction. A**. Ctb73 contains three protein-protein interaction domains; BTB domain, BACK domain and PHR (kelch) domain. **B**. MYC-Ctb73 was immunoprecipitated and probed with anti-HA antibodies to detect binding to LRR1 and LRR5 (upper panel). Lower panels show the expression levels of the HA-LRRs in transfected cells. **C**. MYC-Ctb73 wild-type or deletion mutants were pulled down by anti-MYC antibody and probed against HA (upper panel) to locate LRR5 binding site on Ctb73. Relative expression levels of HA-LRR5 are shown in lower panel. **D**. Two sequential immunoprecipitations were performed on cell lysates expressing FLAG-TEV-Cul3, MYC-Ctb73 and HA-LRR5. FLAG beads brought down Cul3 complexes that were cleaved off the resin using TEV protease. The eluted proteins were verified by Western blot for Cul3. Subsequent immunopreciptiation with MYC antibody brought down a protein complex containing Ctb73. Western blot analysis showed LRR5 binding to the Cul3-Ctb73 complex. Relative protein levels are shown in bottom three panels. **E**. Cell lysates co-expressing Cul3, Ctb73 and LRRs were used in the immunoprecipitation of FLAG-Cul3 that pulled down HA-LRR1 and HA-LRR5. In the same transfection set, immunoprecipitation of FLAG-Cul3 also brought down MYC-Ctb73. Relative expression levels of LRR1, LRR5 and MYC-Ctb73 were verified by Western blotting. See also Additional file 1: Figure S1.

Based on our observation that removal of the BTB binding region of Cul3 enhanced LRR binding we hypothesized that they may either have overlapping binding sites on Cul3 or interact with each other. To test this hypothesis, we examined Ctb73 [18], also called BTBD1 [19,20] (Figure 3A), which is a BTB domain-containing protein previously shown to mediate substrate binding to Cul3, for its ability to bind to LRR1 and LRR5 (Figure 3B). In both cases, Ctb73 bound specifically to the LRRs. To further map the regions of interaction, we deleted the BTB domain on Ctb73. Unexpectedly; this form of Ctb73 showed enhanced binding to the LRRs. We confirmed this enhanced binding with a different combination of LRRs and BTB domain-containing protein mutants (Additional file 1: Figure S1). These results imply that there is a secondary interaction region outside the BTB domain that mediates binding between LRRs and BTB domain-containing proteins.To further dissect the binding interaction between Ctb73 and LRRs, we created several Ctb73 deletion mutants, one of which has a deleted region N-terminal to the BTB domain (Ctb73Δ2-61), which is extremely proline rich (20%) (Figure 3C). We also examined binding between LRR5 and the Ctb73 deletion mutants without the BTB domain, the BACK domain and the Kelch domain. Though each domain deletion failed to completely disrupt the binding, we observed slight reduction between the binding of the Ctb73Δ2-61 mutant and LRRs (Figure 3C, lane 2). We speculated that the reason we could not completely eliminate binding between Ctb73 and LRR5 was that they both were able to bind Cul3 and therefore bridging of Cul3 caused the remaining binding.

### Cul3 forms a complex with a BTB/Kelch domain-containing protein and a LRR domain-containing protein

Based on our observations that LRRs bind to Cul3 and BTB domain-containing proteins, as well as prior knowledge that Cul3 binds BTB domain-containing proteins, we wanted to determine if the three proteins co-existed within the same complex. FLAG-Cul3 with a TEV protease cleavage site (FLAG-TEV-Cul3) was co-expressed with MYC-Ctb73 and HA-LRR5. FLAG-beads were used to purify the Cul3 complex, which were subsequently treated with TEV protease to release the protein complex from the beads. The resulting soluble proteins were subjected to a second round of immunoprecipitation with MYC antibody to pull down MYC-Ctb73. The resulting protein complexes were analyzed by Western blot analysis to detect the presence of the third protein, HA-LRR5. Two sequential affinity purifications of FLAG-Cul3 and MYC-Ctb73 pulled down significant amounts HA-LRR5 only in the presence of all three proteins (Figure 3D, top panel). These data verify our hypothesis that Cul3, a BTB-domain containing protein and an LRR could co-exist within the same complex.Because our results in Figures 2 and 3 suggested a potential for overlapping or co-regulation of binding between BTB domain-containing proteins and LRRs on Cul3, we also compared complex formation on wild type Cul3 versus the mutant that cannot bind BTB domain-containing proteins (FLAG-Cul3Δ51-67). We observed a slight increase in the binding between the LRRs and the Cul3 mutant when compared to wild type (Figure 3E lanes 2 and 4), which further supports a overlapping binding model.

### A B/C-like box sequence mediates LRR binding to Cul3 complexes

**Figure 4:**
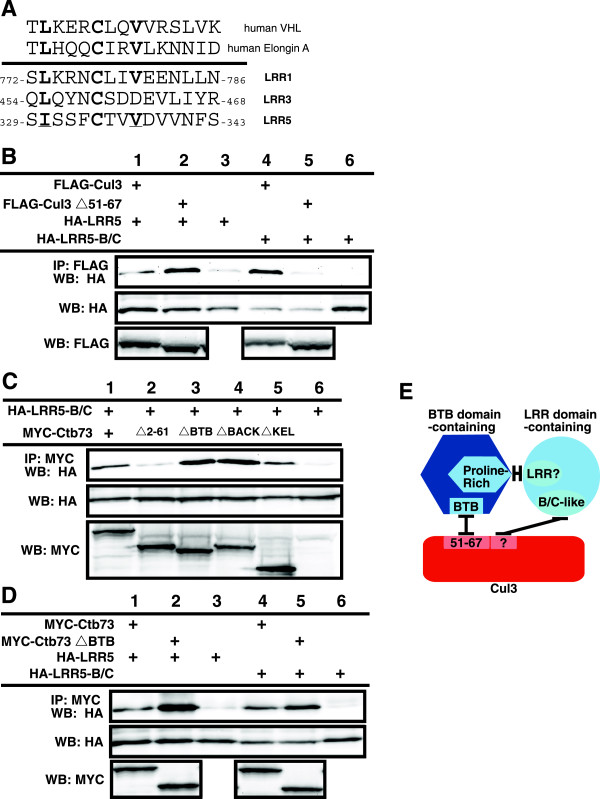
**B/C-like box mediates binding between Cul3 complexes and LRRs. A**. Alignment of VHL and Elongin C B/C boxes with putative B/C-like boxes in three LRRs identified in our study. **B**. Cul3 binding to wild type LRR5 and LRR5-B/C-like box mutant. Upper blot shows immunoprecipitation of Cul3 followed by blotting with anti-HA antibody to detect the presence of LRR5 proteins. Lower blot, relative amounts of LRR5. **C**. Ctb73 binding LRR5-B/C-like box mutants. Upper blot shows immunoprecipitation of Ctb73 followed by blotting with anti-HA antibody to detect the presence of LRR5 protein. Lower blots show relative amounts of LRR5 and Ctb73 proteins. **D**. Binding of Ctb73 and Ctb73ΔBTB mutants to LRR and LRR5-B/C mutant. Upper blot shows immunoprecipitation of Ctb73 followed by blotting with anti-HA antibody to detect the presence of LRR5 proteins. Lower blots show relative amounts of LRR5 and Ctb73 proteins. **E**. Graphical summary of the interactions between Cul3, a BTB domain-containing protein and a LRR domain-containing protein.

As several LRRs are very small and the LRR domain dominates the coding sequence, we began determining the regions on the LRRs that mediate interactions with Cul3 and/or the BTB domain-containing proteins. In this case, the deletion of LRR domain would not be informative because the remaining portion would be short peptides that were unlikely to fold properly. We could not find any conserved regions outside of the LRR domains that were shared by the identified LRR domain containing proteins. Further examination however revealed a sequence adjacent to the LRR domain that strongly resembles a B/C box (Figure 4A). To determine if this sequence may be playing a role in the interaction between the LRR domain containing proteins and Cul3 complexes, we created I330G C334A mutations in the putative B/C-like box of the LRR5 (HA-LRR5-B/C) and tested its binding to Cul3 (Figure 4B) and Ctb73 (Figure 4C). When compared with wild-type LRR5, we observed that the LRR5-B/C mutant showed enhanced binding to wild type Cul3 (Figure 4B, lanes 1 and 4). However, the LRR-B/C interaction with Cul3Δ51-67 mutant that could not bind BTB domain was completely eliminated, though this form of Cul3 mutant showed enhanced binding to wild type LRRs (Figure 4B, lanes 2 and 5). Additionally, the binding between the LRR5-B/C mutant and various Ctb73 deletion mutants were examined. We observed that LRR5-B/C mutant maintained the same binding interaction with wild-type Ctb73 and most of the deletion mutants (Figure 4C). However, the binding interaction between the LRR5-B/C mutant and the Ctb73Δ2-61 was eliminated (Figure 4C, lane2), indicating that this B/C-like box is the major mediator of binding the LRR domain-containing protein to the BTB domain-containing protein. This B/C-like box binds the N-terminal sequence (residues 2–61) on the BTB domain containing protein. In addition, the residual binding between Ctb73Δ2-61 and wild type LRR5 was due to bridging via Cul3. We speculated that the enhanced binding of the LRR-B/C-like box mutant to wild type Cul3 was due to the reduced interaction between the LRR-B/C mutant and a BTB domain-containing protein in the complex, thus eliminating the interaction we have previously shown to regulate LRRs binding to Cul3. Consistent with this model, LRR5-B/C does not show enhanced binding to the version of Ctb73 that is missing its BTB domain, unlike wild-type LRR5 (Figure 4D, lane 2 vs. lane 5). A graphical summary of these interactions is shown in Figure 4E.

### FMOD (LRR5) causes FAZF to become degraded

**Figure 5:**
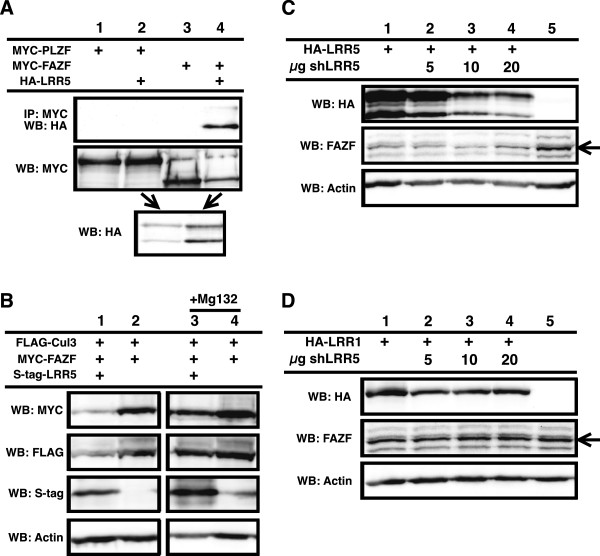
**LRR5 regulates FAZF abundance. A**. LRR5 was co-expressed with either PLZF (lane 2) or FAZF (lane 4). Binding was only seen with the LRR5/FAZF pair (upper blot, lane 4). Protein levels are shown in lower two blots. **B**. Coexpression of LRR5 and FAZF results in reduced FAZF expression (upper blot, lane 1 vs lane 2). Levels of FAZF are restored during co-expression when the proteasome is inhibited by MG-132 (lanes 3 and 4). Relative protein levels are shown in lower blots. **C**. LRR5 expression reduces levels of endogenous FAZF, upper blot shows LRR5 expression with increasing amounts of shRNA. Middle blot is endogenous FAZF (arrow). Lower blot is actin to show relative protein loading levels. **D**. Same as in **C**, but instead of co-expression LRR5, LRR1 was used.

In order to determine the potential cellular role for these complexes that contain a BTB domain-containing protein, an LRR domain-containing protein and Cul3, we performed two hybrid screens using LRR5 as bait. After screening greater than a million clones we identified only one strong binding partner, FAZF. FAZF is a BTB domain-containing protein that has been shown to be important in transcriptional regulation of B-cell differentiation [21]. This was a surprise since we have seen LRR5 bind several BTB domain-containing proteins, and the library we used contains many BTB domain-containing proteins [18]. In order to determine specificity and verify these two proteins bind in mammalian cells LRR5 was co-expressed with either FAZF or the highly similar protein PLZF [22] (Figure 5A). Interestingly, LRR5 bound FAZF and not PLZF and also appeared to cause a reduction in expression levels of FAZF (Figure 5A). We therefore felt that the binding interaction was potentially biologically significant. FAZF overexpression has been shown to cause G1 arrest and apoptosis in B cell lines [21], in addition LRR5 (FMOD) is highly overexpressed in some types of leukemias [23]. Enforced down regulation of LRR5 in these leukemias also results in apoptosis [24]. Based on this observation and on our previous data regarding the relationship between LRR5 binding Cul3 and BTB domain-containing proteins, we postulated that LRR5 might be involved in regulating the levels of the BTB domain-containing protein FAZF. We reasoned that a complex of all three might convert the BTB domain-containing protein to a substrate instead of a substrate adaptor by modulating how it binds to Cul3. Consistent with the proposed hypothesis, the reduced levels of FAZF caused by coexpression of both LRR5 and FAZF could be restored by inhibiting the proteasome with MG-132 (Figure 5B). We then focused on a potential for LRR5 to modulate expression levels of endogenous FAZF using a well-characterized antibody against FAZF [21]. We found that high expression of LRR5 dramatically reduced endogenous levels of FAZF (Figure 5C). As LRR5 levels were decreased using coexpression of shRNA against LRR5, the levels of FAZF increase demonstrating an inverse relationship between expression of LRR5 and FAZF. An analogous experiment in which LRR1 was coexpressed with shRNA against LRR5 showed no change in endogenous FAZF levels (Figure 5D, note: these cells do not express detectable LRR5, not shown). From these data we conclude that LRR5 negatively regulates levels of endogenous FAZF, similar to the regulation we saw with coexpression.

### A method to characterize potential substrates of the BCR ubiquitin ligase complex by mass spectrometry

The identification of known substrates of the BCR ubiquitin ligase complex, such as Mei1 and GluR1, by our MudPIT analysis led to the hypothesis that some of the newly identified Cul3-binding proteins are protein substrates targeted for ubiquitination by the BCR ubiquitin ligase complex. A large number of Cul3-binding proteins in this study made it impossible to individually analyze these potential substrates of the BCR ubiquitin ligase complex. Therefore, we searched for a screening method that would identify a large number of potential substrates in a short period of time. We, and others, have previously observed that Cul3 and protein substrates of the BCR ubiquitin ligase complex could be co-purified by an immunoprecipitation assay [12,25], which led us to speculate that some substrates may remain bound to the Cul3 complex in their ubiquitinated form. In this analysis, purified Cul3 complexes were digested with trypsin, yielding a mixture of ubiquitinated and non-ubiquitinated precursor peptides that were analyzed by the mass spectrometer. Trypsin also cleaved conjugated ubiquitin molecules after the C-terminal lysine 74, leaving two glycine residues attached to the protein substrate on the side chain of the ubiquitinated lysine. Therefore, the mass of a tryptic peptide with a ubiquitinated lysine residue would also include the mass of two glycine residues from the C-terminal end of the conjugated ubiquitin, which would allow us to distinguish these potential substrates of the BCR ubiquitin ligase complex among hundreds of Cul3-binding proteins.

In order to identify potential substrates of the BCR ubiquitin ligase complex, we modified the parameters of the SEQUEST algorithm with a variable modification on lysine of 114 Da, which is consistent with a Gly-Gly modification. This alternative analysis of the mass spectra has led to the identification of 17 potential substrates that were each identified by at least two peptide sequences and spectra with a mass error value of less than 20 ppm and a high Xcorr value (charge 1 ≥ 2, charge 2 ≥ 2.5, and charge 3 ≥ 3) (Table 2). Approximately 27% of the total potential Cul3-binding proteins were conjugated with ubiquitin molecules, only 1.53% of these proteins contain the predicted spectra that matched the mass spectra of both ubiquitin-conjugated and unconjugated peptides (Table 2). Furthermore, the SEQUEST algorithm also distinguishes the position of the modified lysine residues within each precursor peptide. Therefore, this MudPIT approach not only identifies potential substrates of the BCR ubiquitin ligase complex in less time than traditional biochemical assays, it also predicts the ubiquitin conjugation sites on each potential substrate that would otherwise require many mutational analyses to obtain. Based on our analysis, we observed that none of the CLW proteins were identified by this method, thus further supporting our hypothesis that they are not substrates for the ubiquitination process, but rather part of the BCR ubiquitin ligase complex.

**Table 2:** Potential ubiquitin-conjugated Cul3-binding proteins and their ubiquitination sites

**Protein name**	**Number of residues**	**Accession #**	**Domains**	**Peptide sequence**	**Ub-sites**	**Charge state**	**Mass error (ppm)**	**XCorr value**	**Exp L2**	**Exp M3**	**Exp M4**	**Exp M5**	**Exp M6**	**Exp M7**
**Cell division/Cell cycle control**														
Rb1-inducible coiled coil protein 1	1594	NP_055596	No domain	LTHLGTAVSVMAK*	K201	2	12.4	2.012	1	-	-	-	-	-
				TFVQK*EQCDFSNSLK	K828	2	19.7	2.885	-	1	-	-	-	-
**Cytoskeleton**														
DST protein	258	AAH04912	GAS2	K*SPASKLDK	K249	2	15.5	2.055	1	-	-	-	-	-
				SPASKLDK	none	1	18.7	2.136	1	-	-	-	-	-
				EKFILADGASQGMAAFRPR	none	2	16.6	2.693	-	1	-	-	-	-
Filamin B	2591	BAD52434	CH, IG_FLMN	ATQTGDASK	none	1	18.6	1.584	1	-	-	-	-	-
				ETTDFKVDTKAAGSGELGVTMK	none	2	3.9	3.121	-	1	-	-	-	-
				SGCIVNNLAEFTVDPK*	K673	2	9.7	2.139	1	-	-	-	-	-
**Hormone receptor**														
Thyroid hormone receptor interactor 11	1979	NP_004230	No domain	K*VAFDVK*MENEK	K670, K676	2	17.8	2.879	-	-	1	1	2	-
				K*VAFDVKMENEK*	K670, K681	2	5.4	2.581	1	-	-	-	-	-
				MQLLQSLQEQK	none	2	0.6	2.463	1	-	-	-	-	-
**Immune system**														
Lymphocyte antigen 75	1722	NP_002340	FN2, CLECT	DGAICYK*PTK	K1514	2	6.6	2.182	1	-	-	-	-	-
				DGAICYKPTK*	K1517	2	9.8	2.260	1	-	-	-	-	-
**Metabolism**														
Aspartate beta-hydroxylase isoform a	758	NP_004309	Asp-B_Hydr, TPR, Asp-Arg-Hy	ARYGK*AQCEDDLAEK	K381	2	12.1	2.730	-	1	-	-	-	-
				ARYGKAQCEDDLAEK*	K391	2	3.9	2.338	-	2	-	-	-	-
				PKLLNK*FDK	K332	2	3.9	2.338	1	-	-	-	-	-
**Muscle**														
Long myosin light chain kinase	1914	AAQ02673	S_TKc, FN3, IGcam	ESK*LDSLEAAAK	K284	2	5.0	2.382	1	-	-	-	-	-
				ESKLDSLEAAAK*SK*	K293, K295	1	19.7	2.338	1	-	-	-	-	-
Myosin binding protein C, fast type	1141	NP_004524	FN3, IGcam	TSEKK*SDTAGELDFSGLLK*	K175, K185	3	1.4	2.821	1	-	-	-	-	-
				TSENAIVVVAGNK*	K562	2	12.6	2.967	-	-	-	1	-	-
Nesprin-1	8797	AAN60442	CH, SPEC	SAREKGER	none	1	8.9	1.688	1	-	-	-	-	-
				WVQYTAGKQTGIEVKDFGK*	K212	3	20.0	3.426	-	1	-	-	-	-
**Neuron**														
Synaptojanin 2A	1288	AAN73051	Syja_N, IPPc	SSGKIFKDFHEGAINFGPTYK	none	2	0.9	2.005	1	-	-	-	-	-
				TGMGGK*AGNKGAVGIR	K678	2	3.6	2.048	1	-	-	-	-	-
**Oncoprotein**														
BRCA2 and CDKN1A interacting protein	322	CAI12093	No domain	AGLIQSR	none	1	9.2	1.813	1	-	-	-	-	-
				NCEK*SMVEQLDK	K159	1	19.7	1.922	1	-	-	-	-	-
**Signal transduction**														
Endothelial differentiation, sphingolipid G-protein-coupled receptor, 1	382	NP_001391	7tm_1	CPSGDSAGKFK*R	K340	2	6.1	2.170	1	-	-	-	-	-
				HYNYTGK*	K34	1	14.8	1.973	1	-	-	-	-	-
Triple functional domain (PTPRF interacting) variant	2202	BAD92991	SPEC, SH3, RhoGEF, PH_TRIO	AFAAALDER	none	2	15.9	3.027	-	-	1	-	-	-
				LVNASVAFYK*	K1003	2	1.4	2.046	1	-	-	-	-	-
**Ubiquitination**														
Cullin3	768	NP_003581	Cullin	GVKGLTEQEVETILDK*	K414	2	11.9	2.443	1	-	-	-	-	-
**Unknown function**														
Chromosome 10 open reading frame 92	876	AAH34223	No domain	GRKGSIPR	none	1	17.3	1.551	1	-	-	-	-	-
				ARVQTPAVVADSGKSK	none	2	6.7	2.024	1	-	-	-	-	-
				K*GSIPR	K233	1	1.2	1.535	1	-	-	-	-	-
KIAA1602 protein	906	AAH33253	No domain	EGAGGGSPLR	none	2	11.5	2.039	1	-	-	-	-	-
				SKGLPK*	K62	1	3.8	1.931	1	-	-	-	-	-
RING finger and CHY zinc finger domain containing protein 1 variant	263	BAD92309	zf_CHY	DKK*QYHCENCGICR	K65	2	10.2	2.581	-	1	-	-	-	-
				GYRCPLCMHSALDMTR	none	2	1.1	2.126	1	-	-	-	-	-
				PYK*CMHCEK*VFR	K306, K312	2	16.4	2.036	1	-	-	-	-	-

Legend:

*Charge state represents the charge state of the precursor peptide ion.

*Mass error (ppm; parts per million) represents the calculated mass difference between the mass of theoretical precursor peptide and the mass of the experimental precursor peptide.

*Xcorr value represents the calculated value of the accuracy between the peaks of the theoretical spectra and the experimental spectra.

*Ub-sites represents the position of the ubiquitin-conjugated lysine residues in a given protein.

*Number of the peptides based on the corresponding mass spectra from each mass spectrometry experiment shown in Table 1 (L = long gradient; M = MudPIT).

The ubiquitinated lysine residues are marked by an asterisk (*). See also Additional file 1: Figure S2.

To verify this algorithm for predicting potentially real ubiquitination sites, we mutated the predicted site on Cul3 (K414R) based on our MudPIT data indicating that the K414 residue on Cul3 contains a ubiquitination site based on the extra mass that is equivalent to two glycine residues (Table 2). Biochemical analysis demonstrated that K414 was a genuine ubiquitination site for Cul3 in cells (Additional file 1: Figure S2).

## Discussion

Since the discovery of Cul3, the characterization of the BCR ubiquitin ligase has progressed rapidly and has resulted in the identification of protein substrates and novel subunits of the BCR ubiquitin ligase complex [18,26-30]. In this study, a MudPIT approach was chosen for the analysis of Cul3 complexes to identify novel proteins that could be part of the Cul3-dependent ubiquitination pathway. As a result, we have identified new members of the Cul3 complex using two different approaches: (1) the identification of potential substrates of the BCR ubiquitin ligase complex and their ubiquitination sites by altering the criteria in the SEQUEST algorithm to identify Cul3-bound ubiquitin-conjugated proteins, and (2) the analysis of proteins with conserved domains among the MudPIT-identified Cul3-binding proteins that resulted in the characterization of previously unidentified *C*ul3-bound proteins containing either *L*RR domains or *W*D40 domains (CLWs).

Based on our chemical analysis, we propose that the LRR domain-containing proteins (LRRs) function to modify the binding of the BTB domain containing protein to Cul3 such that the BTB domain containing protein becomes a substrate. LRRs bind to Cul3 not only via the BTB domain-containing protein, but also through other regions of the Cul3 complex, as the Cul3 mutant that cannot bind BTB domain-containing proteins (Cul3Δ51-67) can still bind LRRs. In addition, these Cul3-bound LRRs were not ubiquitinated, suggesting they are unlikely to be substrates of the BCR ubiquitin ligase complex. This conclusion is further supported by the fact that substrates are unlikely to share a domain (such as an LRR domain) because the mechanism of substrate recognition is usually based on short sequences [31,32].

### LRR domain-containing proteins regulate binding between Cul3 and BTB-domain containing proteins

We discovered that the LRR domain-containing proteins modulated the binding of the BTB domain-containing proteins binding to Cul3 such that they became substrates instead of substrate adaptors. We found that a region on the BTB domain-containing protein just N-terminal to the BTB domain appears to be mediating the interaction with LRR domain-containing proteins. This region on Ctb73 is extremely proline-rich; it is interesting to note that other BTB domain proteins that have been shown to bind Cul3 also have similar proline rich regions in their N-terminus, like actinfilin, BTBD2, and FAZF (PLZF does not have this region and does not bind LRR5). Thus, this region may play a role in a number of other BTB-domain protein/LRR-domain protein interactions. In addition the LRR domain-containing proteins contain a sequence that resembles a B/C box that mediates binding to the BTB domain-containing proteins.

Based on the identification of the WD40 domain in substrate adaptor subunits of the Cul4 complex, we first proposed that our MudPIT-identified WD40 domain-containing proteins also behave similarly in the Cul3 complex. However, mutational analysis revealed that the interaction site for the WD40 domain-containing proteins lies within the conserved cullin domain in C-terminal region of Cul3, suggesting that their binding interactions did not resemble those found on the LRRs (not shown). Therefore, the WD40 domain-containing proteins may function in a completely different way than the LRRs.

### Potential substrates of the BCR ubiquitin ligase complex identified by a method that involves an altered SEQUEST algorithm

To distinguish novel Cul3-binding proteins from potential substrates of the BCR ubiquitin ligase complex, we adjusted the parameters in the SEQUEST algorithm to recognize the ubiquitin-modified lysine residue in the peptide spectra. Manual validation of the mass spectra revealed that the altered SEQUEST parameters could be used to identify ubiquitinated precursor peptides that are derived from the ubiquitin-conjugated proteins. This technique for the identification of ubiquitin-conjugated proteins using MudPIT mass spectrometry is an approach that can be applied to the analysis of ubiquitinated proteins bound to other ubiquitin ligase complexes. Using this technique, our list of MudPIT-identified Cul3-binding proteins also includes GluR-1, Mei1 and p60/katanin (Additional file 1: Table S1), which are known substrates of the BCR ubiquitin ligase complex [18,33,34], further supporting the validity of this method for substrate identification.

### Cul3 mediated the ubiquitination of lysine residues on its substrates based on the proximity of its location rather than selecting residues based on specific sequences

In this analysis, we have identified the position of lysine residues on which ubiquitin molecules were conjugated to target proteins, as well as the minimum number of ubiquitination sites that are found on each potential substrate. As a heterogeneous population of both ubiquitinated and non-ubiquitinated lysine residues were observed in identical tryptic peptides of Cul3-binding proteins (Table 2), we believe that the BCR ubiquitin ligase complex exhibits some flexibility in the selection of its ubiquitin conjugation site. Furthermore, a significant number of potential substrates also contain more than one ubiquitination site. Additionally, lysine residues that are within 10 residues of each other are often ubiquitinated at both positions. These findings suggest that the mechanism of ubiquitination by the BCR complex is not based on a specific protein sequence adjacent to the ubiquitinated lysine residue, but may be approximated by the distance of the bound substrate from the ubiquitin-conjugating enzyme. This hypothesis is partly based on a study, which indicates that the initial selection for the ubiquitination site on protein substrates is determined by a tertiary complex of E2-E3-substrate [35].

### Model of the BCR^LRR^ complexes

**Figure 6:**
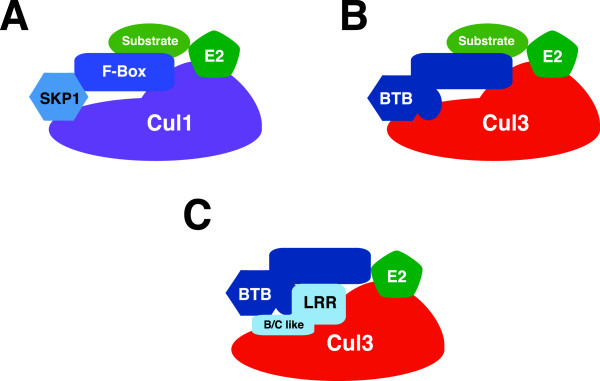
**A proposed novel regulatory model for the Cul3 complex. A**. A schematic diagram of the SCF complex demonstrates that the N-terminal domain of Cul1 is bound to Skp1 protein and a substrate adaptor F-box-containing protein. **B**. The current model of the BCR complex proposed that a BTB domain-containing protein utilizes other protein-protein interacting domains to bind its protein substrates. **C**. A proposed model for an alternative BCR ubiquitin ligase complex. In this case, we propose that the LRRs bind to Cul3 as well as to a unique region on the BTB domain-containing protein via a B/C-like box. This binding serves to change the binding position of the BTB domain-containing protein on Cul3 causing it to be a substrate instead of a substrate adaptor protein.

The SCF model for cullin-based E3 ligase stated that the cullin provides a scaffold to assemble a ubiquitin-conjugating enzyme (E2), Skp1 and an F-box containing protein. The F-box containing protein recognizes substrates and recruits them to the E3 complex (Figure 6A). Previous models of the BCR ubiquitin ligase complex proposed that a BTB domain-containing protein serves the combined functions of both Skp1 and the F-box protein by binding to Cul3 via its BTB domain and using its secondary protein-protein interacting domain to target its bound substrates for ubiquitination (Figure 6B). This model is supported by the identification of substrates of the Cul3 ubiquitin ligase complex and their adaptor proteins, such as Nrf2 and Keap1, GluR1 and actinfillin, Dishevelled and KLHL12, Aurora B and KLHL11/KLHL9 and p60/katanin and KLHDC5 [18,36-39]. In this report, we have identified an additional group of Cul3-binding proteins that contain either LRR domains or WD40 domains. Incidentally, these some of these LRR domain-containing proteins also interact with the BTB domain-containing proteins. We propose that the B/C-like box domain on the LRRs and a unique, proline rich domain on the BTB domain containing protein Ctb73 is responsible for the binding between the BTB domain-containing protein, the LRRs and Cul3. This binding causes the BTB domain-containing protein to become a substrate, possibly by changing how it binds to Cul3 (Figure 6C).

## Conclusions

In this report, we have identified an additional group of Cul3-binding proteins that contain either LRR domains or WD40 domains. We propose that the B/C-like box domain on the LRR domain-containing proteins and a unique, proline rich domain on the BTB domain containing protein Ctb73 is responsible for the binding between the BTB domain-containing protein, the LRRs and Cul3. However, the binding interaction may be more complex because deletion of the BTB domain resulted in enhanced LRR binding to Cul3. These observations led us to further investigate these interactions. We found that one of these LRR domain-containing proteins, LRR5/FMOD, causes a BTB domain-containing protein, FAZF, to become a substrate for the BCR complex. Possibly, this mechanism is a way for BTB domain proteins to be removed from substrates. There are several know examples of cullin based E3 ligases degrading their substrate adaptors [40,41], thus we suggest that this may be a general theme for the function of these novel Cul3 binding proteins. The selective specificity of LRR5 for FAZF and not the highly homologous protein PLZF is intriguing and may indicate specificity for LRR domain-containing proteins binding BTB domain-containing proteins. Additional biochemical analyses will lead to a more thorough understanding of this novel regulatory mechanism.

## Methods

### Plasmids

Full-length cDNA sequences of Cul3, BTBs, and CLWs were cloned from a human testis cDNA library and inserted in-frame into the 3XFLAG-24 (Sigma-Aldrich), CS2 + MT, and CS2 + HA vectors, respectively. All clones were fully sequenced. Mutations in the Cul3 protein were made using the QuikChange site-directed mutagenesis kit (Stratagene). All plasmids were sequenced after mutagenesis. Expression plasmids for MudPIT analysis were constructed by cloning the full-length Cul3 sequence into the 3XFLAGNEO-26 plasmid (Sigma-Aldrich) in-frame with the FLAG tag. A TEV recognition sequence and a calmodulin-binding protein sequence were inserted in-frame between the 3XFLAG epitope and the Cul3 sequence, creating the 3XFLAG-TEV-CBP-Cul3 plasmid that was used for tandem affinity purification. As a control, a 3XFLAG-TEV-CBP-actin plasmid was created by cloning the full-length actin sequence from a human testis cDNA library and inserting it in-frame into the 3XFLAG-TEV-CBP plasmid.

### Cell culture and protein expression

HEK293 cell lines were maintained in DMEM media supplemented with 10% fetal bovine serum (FBS). For transient protein expression, HEK293 cells were transfected with either 3XFLAG-TEV-CBP-Cul3 or 3XFLAG-TEV-CBP-actin plasmids as described previously [42].

### Two-hybrid screening

pGildaLRR5 (FMOD) was screened using the Matchmaker LexA two hybrid system (Clontech). A human testis library was used and 2.7 million clones were screened. The only interacting clone identified was FAZF.

### Immunoblotting, immunoprecipitation and immunofluorescence

Transfected HEK293 cells were harvested in 2.7 mM EDTA in phosphate-buffered saline and cell lysates were prepared by sonication for 10 seconds in radioimmunoprecipitation assay (RIPA) buffer (1% NP-40, 1% sodium deoxycholate, 0.1% SDS, 150 mM NaCl, 0.01 M sodium phosphate buffer pH 7.2 and 2 mM EDTA). For immunoprecipitation assays, the clarified lysates were incubated with primary antibody, followed by protein A-Sepharose beads for 1 hr each at 4°C. Beads were spun down and washed twice with RIPA buffer. Protein samples were separated by electrophoresis on 10% polyacrylamide gels, transferred to PVDF membranes, and incubated overnight in primary antibodies. Proteins were visualized using enhanced chemiluminescence followed by digital acquisition using an AlphaInnotech Fluorochem SP system. The following antibodies were used in these experiments: anti-FLAG M2 monoclonal antibody (Sigma-Aldrich), anti-actin polyclonal antibody (Sigma-Aldrich), anti-HA monoclonal antibody (Covance), anti-MYC sc-789 polyclonal antibody (Santa Cruz Biotechnology), and anti-Cul3 antibody which has been previously described [43]. The anti-FAZF antibody was a kind gift of Dr. Maureen Hoatlin (OHSU). Immunofluorescence was performed as previously described [18].

### FLAG immunoprecipitation and TEV protease cleavage

Clarified cell lysates were incubated with anti-FLAG M2 agarose slurry (Sigma-Aldrich) at 4°C for 1 hour. The protein-bound beads were washed twice in RIPA buffer and three times in tobacco etch virus (TEV) buffer (10 mM HEPES-KOH pH 8.0, 150 mM NaCl, 0.1% NP-40, 0.5 mM EDTA and 1 mM DTT). The FLAG beads were then incubated with the AcTEV protease enzyme (Life Technologies) in TEV buffer overnight at 4°C with constant agitation. After TEV cleavage was complete, the supernatant containing Cul3 complexes was collected and the FLAG beads were washed three times with TEV protease buffer and twice with calmodulin-binding buffer (10 mM 2-mercaptoethanol, 10 mM HEPES-KOH pH 8.0, 150 mM NaCl, 1 mM MgOAc, 1 mM Imidazole, 0.1% NP-40, and 2 mM CaCl_2_) to recover the Cul3 complexes that were cleaved but remained bound to the beads. A sample of FLAG beads prior to TEV cleavage and protein samples from each eluted fraction were immunoblotted and probed with the appropriate antibodies to determine the efficiency of TEV cleavage and to estimate the protein yield for the next purification step.

### Purification with calmodulin-conjugated beads

Collected protein samples from the FLAG immunoprecipitation and the TEV protease cleavage were supplemented with 1 M CaCl_2_ solution at a 25:1 ratio, followed by incubation with Calmodulin Sepharose™ 4B beads (Amersham Biosciences) for 90 minutes at 4°C. The beads were spun down at 750 × g and washed three times with the calmodulin-binding buffer and twice with the calmodulin-rinsing buffer (50 mM NH_4_HCO_3_ pH 8.0, 75 mM NaCl, 1 mM MgOAc, 1 mM imidazole, and 2 mM CaCl_2_). The bound protein complexes were then eluted from the calmodulin beads using calmodulin-eluting buffer (50 mM ammonium bicarbonate, pH 8.0 and 25 mM EGTA) and collected in separate fractions. A sample of protein-bound calmodulin beads and protein samples of each eluted fraction were immunoblotted and probed with the appropriate antibodies to estimate the relative protein yield of each eluted fraction. The fractions with the most amount of protein were selected for further analysis using MudPIT techonology.

### Trypsin digestion and peptide desalting

An equal volume of 8 M urea, pH 8.3, was added to the eluants from the calmodulin bead purification and the mixture was denatured at 95°C for 5 minutes. After the mixture had cooled to room temperature, trypsin (Promega) was added to purified proteins in a 1:40 w/w ratio and the mixture was incubated overnight at 37°C. Once the digestion was complete, the peptide mixture was diluted with 0.1% acetic acid, adjusted to pH 3, and then loaded onto a C18 reverse phase peptide macrotrap cartridge (Michrom Bioresources) that had been equilibrated with 0.1% acetic acid. The cartridge with the bound protein was washed twice with 0.1% acetic acid and the peptides were eluted with 70% acetonitrile in 0.1% acetic acid. The peptide mixture was dried using a SAVANT SC110A SpeedVac centrifuge with an RVT400 refrigerated vapor trap (Thermo Electron Corp.) and stored at -80°C.

### Strong cation exchange chromatography

The dried peptides were reconstituted in 0.1% acetic acid to a concentration of 0.5 mg/ml. They were subsequently loaded onto a strong cation capillary column (home-packed on a pressure bomb) with an inner diameter of 247.0 μm (Polymicro technology), 20 cm in length, and fritted by M-520 (Upchurch) containing polysulfoethyl A™ bulk material of 5 μm diameter and 300 Å pore size (The Nest Group, Inc.). The samples were eluted with a 12-step salt gradient between buffer C (30% acetonitrile, 5 mM phosphate buffer pH 3) and buffer D (30% acetonitrile, 5 mM phosphate buffer pH 3, and 500 mM ammonium acetate) with 0.01 ml/min flow rate for 10 minutes in each gradient step. Each solution step consists of a different ratio of buffer C to buffer D (C/D): step 1 = 100/0, step 2 = 98/2, step 3 = 96/4, step 4 = 94/6, step 5 = 92/8, step 6 = 90/10, step 7 = 88/12, step 8 = 86/14, step 9 = 84/16, step 10 = 80/20, step 11 = 50/50, and step 12 = 0/100. The eluted peptides from each salt gradient step were collected separately, dried and stored at -80°C until the next step.

### On-line reverse phase column and tandem mass spectrometry

In preparation for analysis by mass spectrometry, the dried peptide samples from each salt step fraction were reconstituted in 0.1% acetic acid. The peptides were loaded onto a pre-column (360 μm outer diameter × 75 μm inner diameter) containing 2 cm of 5 μm Monitor C18 resin (Column Engineering). They were then eluted into the mass spectrometer (LTQ-FTICR, Thermo Electron Corp.) through an analytical column (360 μm outer diameter × 75 μm inner diameter fused silica packed with 12 cm of 5 μm Monitor C18 particles with an integrated ESI emitter tip having a 4 μm opening fritted with 3 μm silica particles; Bangs Labs, Fishers, IN) by using a linear HPLC gradient ranging from 0-70% acetonitrile over 30 minutes using buffer A (0.1 M acetic acid) and buffer B (0.1 M acetic acid in acetonitrile) with peak parting. The eluted peptides were then processed by an LTQ-FTICR mass spectrometer (data-dependent scanning with 1 MS scan followed by 5 MS/MS scans). MS/MS spectra were generated and automatically searched against the human NCBI non-redundant protein database using the SEQUEST algorithm provided with Bioworks 3.2 SR (Thermo Electron Corp.). The list of predicted proteins was imported into a custom-made FileMaker Pro relational database for data analysis.

### Data analysis and domain prediction

MS/MS spectra with mass error of less than 20 ppm and high Xcorr value (charge 1 ≥ 2, charge 2 ≥ 2.5, and charge 3 ≥ 3) were chosen for further analysis. The exact predicted peptide sequences were searched against sequences in the NCBI protein-protein database to uncover potential Cul3-binding proteins [44]. All corresponding protein sequences were then scanned for predicted domains with the NCBI conserved domain architecture retrieval tool (CDART) [45]. MudPIT mass spectrometry was also performed on tandem affinity purified actin complexes to eliminate proteins that may be mistaken for binding partners. These may be contaminants from the purification that bind non-specifically to the CBP tag or to the surface of large globular proteins, such as actin. Predicted protein candidates that were identified in both the actin and the Cul3 complexes were regarded as false positives and removed from the list of potential Cul3-binding proteins. The remaining proteins were then categorized based on their conserved domains and their roles in cellular pathways.

### Availability of supporting data

The data sets supporting the results of this article are included within the article (and its additional supplemental files).

## Abbreviations

BACK: BTB and C-terminal Kelch; BCR: BTB-Cul3-Rbx1; BPB: β-propeller domain; BTB: Broad-Complex, Tramtrack and Bric a brac; CAND1: Cullin-associated and neddylation-dissociated 1; CBP: Calmodulin-binding protein; CLW: Cul3-bound proteins that contain either the LRR or WD40 domain; Ctb: Cul3-binding protein; DDB1: DNA damage-binding protein1; DWD: DDB1 binding WD40; ECS: Elongin-Cul2-SOCS-box; Gly: Glycine; KLHL5: Kelch-like family member 5; LC/MS-MS: Liquid chromatography-tandem mass spectrometry; LRR: Leucine-rich repeat; Mei1: Meiosis inhibitor 1; MudPIT: Multidimensional Protein Identification Technology; Nedd8: Neural precursor cell expressed; Rbx1: RING-box protein 1; Rpn: Ribophorin; SCF: Skp1-Cullin-F-box; Skp1: S-phase kinase-associated protein 1; Skp2: S-phase kinase-associated protein 2, SOCS, suppressors of cytokine signaling; STIP1: Stress-induced-phosphoprotein 1; TAP: Tandem affinity purification, TEV, Tobacco etch virus.

## Competing interests

The authors declare that they have no competing interests.

## Authors’ contributions

WW did the cloning, expressed the Cul3 complexes, performed tandem affinity purification and immunoprecipitation (results shown in Figures 1, 6, Additional file 3: Figure S2, Table S3 and Additional file 4: Table S4), and wrote the manuscript. MW performed experiments shown in Figures 2, 3 and 4. BD performed experiments shown in Figure 5. KY performed the LC/MS-MS and analyzed the data using the SEQUEST algorithm, as shown in Additional file 1: Table S1 and Additional file 2: Table S2. AS conceived the idea about altering the SEQUEST algorithm to select for substrates of the BCR ubiquitin ligase complex. JDS conceived the study. All authors read and approved the final manuscript.

## Supplementary Material

Additional file 1: Table S1 Potential Cul3-binding proteins that were identified by MudPIT. Click here for file

Additional file 2: Table S2 Potential Cul3-binding proteins that were identified by one peptide sequence from MudPIT analysis. Click here for file

Additional file 3: Table S3 Summary of conserved domains of potential Cul3 and actin-binding proteins. **Figure S1.** LRR3 binds Ctb62. HA-tagged LRR3 was transfected into HEK293 cells, either alone (third lane) or with MYC-tagged Ctb62 (first lane) or with MYC-tagged Ctb62 deleted for its BTB domain. Lower gel shows an immunoblot of levels of expression of LRR3 and the upper blot shows binding of LRR3 to Ctb62 by immunoprecipitating Ctb62 followed by an immunoblot for LRR3. **Figure S2.** Cul3 is ubiquitinated at the lysine 414 residue. HEK293 cells were transfected with vectors expressing wild-type Cul3, Cul3K414R mutant, and HA-tagged ubiquitin. Lysates were prepared, checked for protein expression (bottom), and immunoprecipitated with anti-HA antibody. The precipitates were separated by SDS-PAGE and analyzed by immunoblot for Cul3. Click here for file

Additional file 4: Table S4 Summary of proteins that contain conserved domains of interest from MudPIT-identified Cul3-binding proteins. Click here for file
