# Supplementary material for: Novel Cul3 binding proteins function to remodel E3 ligase complexes
Source: BMC Cell Biol. 2014 Jul 10;15:28. doi: 10.1186/1471-2121-15-28 (PMC4107866; doi:10.1186/1471-2121-15-28)
Supplement: Additional file 3: Table S3 — Summary of conserved domains of potential Cul3 and actin-binding proteins. Figure S1. LRR3 binds Ctb62. HA-tagged LRR3 was transfected into HEK293 cells, either alone (third lane) or with MYC-tagged Ctb62 (first lane) or with MYC-tagged Ctb62 deleted for its BTB domain. Lower gel shows an immunoblot of levels of expression of LRR3 and the upper blot shows binding of LRR3 to Ctb62 by immunoprecipitating Ctb62 followed by an immunoblot for LRR3. Figure S2. Cul3 is ubiquitinated at the lysine 414 residue. HEK293 cells were transfected with vectors expressing wild-type Cul3, Cul3K414R mutant, and HA-tagged ubiquitin. Lysates were prepared, checked for protein expression (bottom), and immunoprecipitated with anti-HA antibody. The precipitates were separated by SDS-PAGE and analyzed by immunoblot for Cul3. [file 1471-2121-15-28-S3.pdf]

Figure S1.

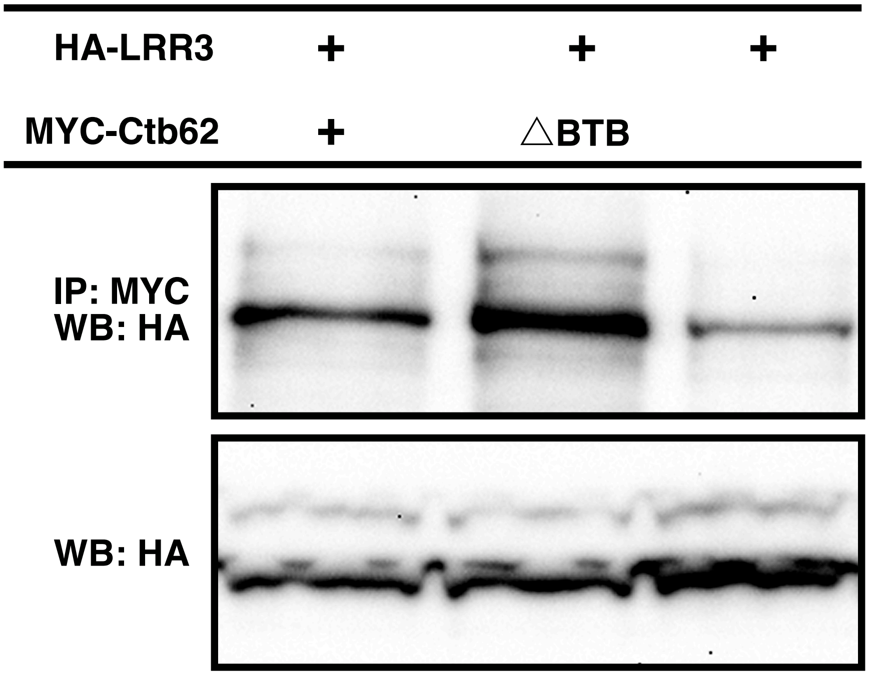

Figure S2.

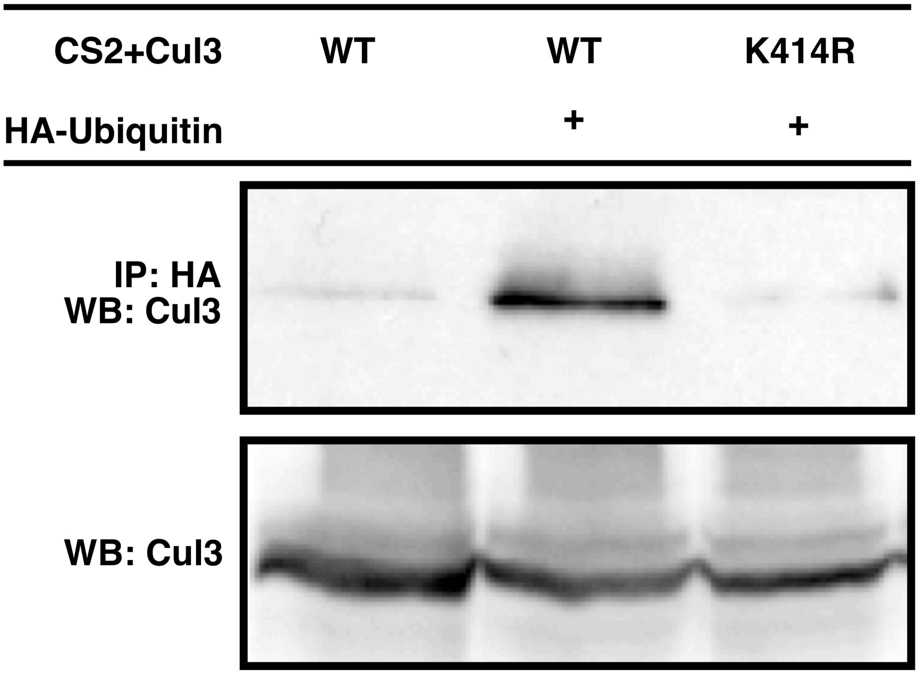

Table S3. Summary of conserved domains of potential Cul3 and actin-binding proteins.

Domain	Cul3-binding proteins			Actin binding proteins		
	Number of Proteins	Percent of Total Proteins	Protein Enrichment*	Number of Proteins	Percent of Total Proteins	Protein Enrichment*
Armadillo	4	0.50	50.0	4	0.60	50.0
BTB	4	0.50	40.0	6	0.94	60.0
Homeobox	9	1.10	69.2	4	0.60	30.8
KRAB	11	1.38	61.1	7	1.09	38.9
LRR	10	1.25	90.9	1	0.15	9.1
PDZ	6	0.75	37.5	10	1.56	62.5
RING	6	0.75	66.7	3	0.47	33.3
Serine/Threonine Kinase	10	1.25	43.5	13	2.03	56.5
WD40	22	2.76	73.3	8	1.25	26.6
Zinc Finger	26	3.26	63.4	15	2.35	36.6
Unclassified	243	30.5	56.9	184	28.8	43.1
Total Number of Proteins	796	100		638	100	

* Percent enrichment of Cul3- or actin-binding proteins with a particular domain compared to the total number of the MudPIT-identified proteins that contains the same domain.

* Percent enrichment of Cul3-binding proteins = Number of Cul3-binding proteins / (Cul3-binding proteins + actin-binding proteins) x 100.

SUPPLEMENTARY FIGURE AND TABLE LEGENDS

Figure S1. LRR3 binds Ctb62. HA-tagged LRR3 was transfected into HEK293 cells, either alone (third lane) or with MYC-tagged Ctb62 (first lane) or with MYC-tagged Ctb62 deleted for its BTB domain. Lower gel shows an immunoblot of levels of expression of LRR3 and the upper blot shows binding of LRR3 to Ctb62 by immunoprecipitating Ctb62 followed by an immunoblot for LRR3.

Figure S2. Cul3 is ubiquitinated at the lysine 414 residue. HEK293 cells were transfected with vectors expressing wild-type Cul3, Cul3K414R mutant, and HA-tagged ubiquitin. Lysates were prepared, checked for protein expression (bottom), and immunoprecipitated with anti-HA antibody. The precipitates were separated by SDS-PAGE and analyzed by immunoblot for Cul3.

Table S1. Potential Cul3-binding proteins that were identified by MudPIT.

Table S2. Potential Cul3-binding proteins that were identified by one peptide sequence from MudPIT analysis.

Table S3. Summary of conserved domains of potential Cul3 and actin-binding proteins.

Table S4. Summary of proteins that contain conserved domains of interest from MudPIT-identified Cul3-binding proteins.
